# Capture Sequencing Enables Sensitive Detection of Tick-Borne Agents in Human Blood

**DOI:** 10.3389/fmicb.2022.837621

**Published:** 2022-03-07

**Authors:** Santiago Sanchez-Vicente, Komal Jain, Teresa Tagliafierro, Alper Gokden, Vishal Kapoor, Cheng Guo, Elizabeth J. Horn, W. Ian Lipkin, Rafal Tokarz

**Affiliations:** ^1^Center for Infection and Immunity, Mailman School of Public Health, Columbia University, New York City, NY, United States; ^2^Lyme Disease Biobank, Portland, OR, United States; ^3^Department of Epidemiology, Mailman School of Public Health, Columbia University, New York City, NY, United States

**Keywords:** tick-borne agents, TBDCapSeq, next generation sequencing, *Borrelia*, *Babesia*, Lyme disease, babesiosis

## Abstract

Assay sensitivity can be a limiting factor in the use of PCR as a tool for the detection of tick-borne pathogens in blood. We evaluated the performance of Tick-borne disease Capture Sequencing Assay (TBDCapSeq), a capture sequencing assay targeting tick-borne agents, to test 158 whole blood specimens obtained from the Lyme Disease Biobank. These included samples from 98 individuals with signs and symptoms of acute Lyme disease, 25 healthy individuals residing in Lyme disease endemic areas, and 35 samples collected from patients admitted to the Massachusetts General Hospital or referred to the infectious disease clinic. Compared to PCR, TBDCapSeq had better sensitivity and could identify infections with a wider range of tick-borne agents. TBDCapSeq identified a higher rate of samples positive for *Borrelia burgdorferi* (8 vs. 1 by PCR) and *Babesia microti* (26 vs. 15 by PCR). TBDCapSeq also identified previously unknown infections with *Borrelia miyamotoi*, *Ehrlichia*, and *Rickettsia* species. Overall, TBDCapSeq identified a pathogen in 43 samples vs. 23 using PCR, with four co-infections detected versus zero by PCR. We conclude that capture sequencing enables superior detection of tick-borne agents relative to PCR.

## Introduction

Polymerase chain reaction (PCR) is the primary molecular assay used for clinical diagnosis of tick-borne diseases (TBD). The sensitivity of PCR can vary depending on the agent targeted, the sample type, and the timing of specimen collection (early vs. late infection; Brettschneider et al., 1998; Brouqui et al., 2004). Blood is the most frequent specimen used in molecular testing (Hu, 2018). PCR of whole blood is highly useful for diagnosis of acute anaplasmosis and babesiosis (Sanchez et al., 2016). Patients who are culture positive for *Anaplasma phagocytophilum* have PCR sensitivities between 77 and 80%, whereas in patients who have a positive blood smear for *Babesia microti* the sensitivity level increases up to 100% (Wormser et al., 2013; Wang et al., 2015). However, PCR is not typically used for detection of *Borrelia burgdorferi* because of low pathogen burden coupled with transient spirochetemia in blood (Benach et al., 1983; Aguero-Rosenfeld et al., 2005). Another challenge is the detection of possible co-infections. Although multiplex PCR assays have been implemented, these assays do not test for the whole breadth of pathogens, and most diagnostic commercial assays still test for only a single agent (Pritt et al., 2011; Chan et al., 2013; Buchan et al., 2019; Modarelli et al., 2019; Shakir et al., 2020; Galaxydx, 2021).

Unbiased next generation sequencing (UNGS) methods have advantages over PCR for molecular detection of microbial pathogens. Whereas PCR requires precision in matching the sequences of primers and reporter oligonucleotides with those of templates, there is no such requirement with UNGS, where random probes facilitate the amplification of all nucleic acids in the sample. Therefore, UNGS has the potential to simultaneously detect the whole spectrum of agents, and to identify novel species or strains (Schlaberg et al., 2017; Hong et al., 2018; Samarkos et al., 2018; Blauwkamp et al., 2019; Gu et al., 2019; Miller et al., 2019). Although PCR has advantages in sensitivity, cost, and simplicity, improvements in next generation sequencing (NGS) such as multiple dual index barcoding and streamlined bioinformatic analysis pipelines have reduced operating costs. Capture sequencing is a NGS method that provides additional advantages over both PCR and UNGS. This approach uses agent-specific probes to selectively capture the template of interest prior to sequencing (Briese et al., 2015; Allicock et al., 2018). Capture sequencing results in a substantial increase in sensitivity surpassing quantitative PCR. The utility of this approach has been documented for viral and bacterial agents, and with the recent development of the TBD Capture Sequencing Assay (TBDCapSeq), we have demonstrated its potential for detection of tick-borne pathogens (Jain et al., 2021). In this study, we examined the utility of TBDCapSeq as a tool for molecular detection of tick-borne agents in human blood with a focus on specimens from patients with early localized Lyme disease.

## Materials and Methods

### Samples

Our study consisted of 158 whole blood specimens obtained from the Lyme Disease Biobank sample repository (Table 1; Horn et al., 2020). This repository includes cohorts of well-characterized samples collected from patients with Lyme disease and healthy controls from TBD endemic areas (Northeast and Upper Midwest). All samples were collected between 2014 and 2019. We examined 98 samples from individuals enrolled in community settings with signs and symptoms of acute Lyme disease (designated as early cohort). Within this cohort, type 1 samples (*n* = 48) were defined by the presence of erythema migrans (EM) skin lesions of >5 cm. Type 2 samples (*n* = 50) had suspected tick exposure and clinical symptoms suggestive of Lyme disease but no EM lesions. Twenty-five samples were collected to represent healthy individuals residing in Lyme disease endemic areas with no history of Lyme disease or other tick-borne infections. All samples were tested by qPCR for multiple tick-borne pathogens and serology for antibodies to *B. burgdorferi* (Horn et al., 2020). We also tested 35 samples collected from patients admitted to Massachusetts General Hospital (MGH) or referred to the infectious disease clinic. These consisted of a mix of samples positive by PCR for tick-borne infections and matched ill controls that presented with fever, high LFTs, and often low platelets. All samples were analyzed at Columbia University in a blinded fashion. Positive calls were reported to the Lyme Disease Biobank at which time the clinical and laboratory data for the specimens were provided.

**Table 1:** Comparison of the results of Tick-borne disease Capture Sequencing Assay (TBDCapSeq) to PCR.

	Lyme disease biobank qPCR					Columbia multiplex qPCR					TBDCapSeq				
Agent	Early cohort*^a^*		Healthy controls*^b^*	MGH*^c^*	Total	Early cohort		Healthy controls	MGH	Total	Early cohort		Healthy controls	MGH	Total
	Type 1	Type 2				Type 1	Type 2				Type 1	Type 2			
*Anaplasma phagocytophilum*	1	2	0	3	6	1	3	0	3	7	1	3	0	3	7
*Babesia microti*	8	2	0	5	15	8	2	0	5	15	11	5	0	10	26
*Borrelia miyamotoi*	0	0	0	0	0	0	0	0	0	0	0	1	0	0	1
*Borrelia burgdorferi*	1	0	0	0	1	1	0	0	0	1	5	2	0	1	8
*Ehrlichia chaffeensis*	ND*^d^*	ND	ND	ND	ND	ND	ND	ND	ND	ND	0	2	0	0	2
*Ehrlichia ewingii*	ND	ND	ND	ND	ND	ND	ND	ND	ND	ND	0	1	0	0	1
*Rickettsia rickettsii*	ND	ND	ND	ND	ND	ND	ND	ND	ND	ND	0	0	0	1	1
*Rickettsia felis*	ND	ND	ND	ND	ND	ND	ND	ND	ND	ND	1	0	0	0	1

a Type 1 *n* = 48; Type 2 *n* = 50.

b Healthy controls *n* = 25.

c Massachusetts General Hospital (MGH) *n* = 35.

d ND, Not done.

### TBDCapSeq Probe Set

We employed an updated TBDCapSeq probe set that included oligos targeting the complete genomes of 14 primary tick-borne agents found in the United States. The targets included *B. burgdorferi*, *Borrelia mayonii*, *Borrelia miyamotoi*, *Babesia microti*, *Anaplasma phagocytophilum*, *Ehrlichia muris eauclaireensis*, *Ehrlichia chaffeensis*, *Rickettsia parkeri*, *Rickettsia rickettsii*, and *Francisella tularensis*, Powassan virus, Heartland virus, Colorado tick fever virus, and Bourbon virus (Jain et al., 2021). For *B. burgdorferi*, we targeted the genomes of three distinct genotypes (strains B31, N40, and 297). The probes were designed to the chromosome and all plasmids. In addition, we included probes for 27 different *ospC* alleles (OspC types A–T). The final set consisted of approximately 500,000 probes.

### NGS Sequencing and Capture Methods

DNA from whole blood was extracted using 400 μl of each sample with the QIAamp DNA Blood Mini Kit (Qiagen) and eluted in 50 μl of water. Samples were tested in parallel by TBDCapSeq and qPCR using our own in-house multiplex PCR assay targeting *A. phagocytophilum*, *B. burgdorferi*, *B. miyamotoi*, and *B. microti* using 5 μl of template DNA per assay (Tokarz et al., 2017). For TBDCapSeq analyses, samples were pooled (five pools, *N* = 34, 21, 35, 35, and 34 per pool). DNA concentrations were measured with the Qubit High Sensitivity Double-stranded DNA kit and Qubit 2.0 Fluorometer (Invitrogen). Libraries with custom dual-indexes were prepared with the Kapa Hyperplus kit (Roche) using 25–50 ng of input material and the recommended adaptor concentrations and cycling parameters. Amplified libraries were quantified on a TapeStation 4200 using the D1000 kit (Agilent Technologies). Measured DNA concentrations were used to equally pool libraries. After quantification on the TapeStation 4200, 1 μg of the pool was mixed with 5 μg of COT Human DNA (Thermo Fisher Scientific) and 2,000 pmol of Blocking Oligo pool (Roche). The mixture was fully dehydrated at 60°C in a vacuum centrifuge. The dried pool was resuspended in 7.5 μl Hybridization Buffer and 3 μl Hybridization Component A (Roche) to a volume of 10.5 μl and heated at 95°C for 5 min before the addition of 4.5 μl of custom biotinylated TBD SeqCap EZ Probe pool (Roche). The mixture was again heated at 95°C for 5 min before being incubated at 47°C for 16–20 h. After incubation, the probes were pulled down using magnetic streptavidin SeqCap Capture beads (Roche) and washed with buffers of decreasing stringency (SeqCap EZ Hybridization and Wash Kit, Roche). The probe-bound DNA was eluted in water and amplified by 16 cycles of PCR using Illumina universal primers (Kapa HiFi HotStart Ready Mix, Roche) using Illumina Universal Primers. Finally, the amplified pool was quantified (Agilent Tapestation) and sequenced on an Illumina NextSeq2000 platform that generated 150 nt long single end reads. For bioinformatics analyses, we followed our standard pipeline for agent identification (Briese et al., 2015; Tokarz et al., 2019).

Following the analysis of all 158 specimens, we selected 31 samples for resequencing to evaluate assay reproducibility. These included healthy controls and an assortment of samples found to be low-positive and negative by TBD-CapSeq. For each sample, new sequencing libraries were prepared. Three samples were retested in duplicate, each with two different library preparations. The pool (*N* = 34) was then subjected to capture sequencing as outlined above. For direct comparison of TBDCapSeq to UNGS, we selected another set of samples (*N* = 35) that included healthy controls, and TBD-CapSeq low-positives and negatives. New sequencing libraries were prepared for each sample and examined using UNGS.

### Threshold Establishment

We established stringent threshold criteria required for a sample to be called positive. These criteria were based on our past experience with capture sequencing as well as TBDCapSeq analyses of control specimens in the current and in our previous studies (Briese et al., 2015; Allicock et al., 2018; Tokarz et al., 2018). First, we normalized the agent-specific reads with the number of primer-trimmed reads, and only the samples that generated at least one agent-specific read per 1 million primer-trimmed reads were analyzed further. The reads could originate from any part of the genome. For bacterial pathogens, our threshold for a read to be identified as positive required a length of ≥100 nt and sequence identity of ≥98% by BlastN. In addition, we required the presence of reads mapping to five different genetic loci (separated by a minimum of 100 nt). We established a higher threshold for *B. microti*, requiring the presence of reads from ≥15 loci from each of the four chromosomes of *B. microti*.

## Results

The DNA from each sample was analyzed using multiplex qPCR and TBDCapSeq. The results were then compared to the PCR tests performed after participant enrollment (Table 1). The results of both qPCR analyses were 96% congruent. The lone exception was a sample positive in our qPCR assay for *A. phagocytophilum* that was negative in the original test. Both qPCR tests detected *B. burgdorferi* in only a single sample from the early cohort (EC), with *B. microti* (*N* = 10 in EC, *N* = 5 in MGH) and *A. phagocytophilum* (*N* = 4 in EC, *N* = 3 in MGH) more frequently detected.

For TBDCapSeq analysis, samples were grouped into five pools (Table 2). Illumina sequencing generated between 532 and 369 million reads per pool. All PCR-positive samples (*N* = 23) were identified by TBDCapSeq (Table 1). In addition, we identified 11 additional samples that were positive for *B. microti* and seven samples that were positive for *B. burgdorferi* by TBDCapSeq. Two of these samples were positive for both *B. burgdorferi* and *B. microti*. We detected two other co-infections, one with *A. phagocytophilum* and *B. microti*, and one with *Rickettsia felis* and *B. microti*. We also detected *B. miyamotoi* in one sample that was not detected by PCR. TBDCapSeq also identified five samples that contained sequences of agents not transmitted by *Ixodes scapularis*. Three samples were positive for *Ehrlichia* (two for *E. chaffeensis* and one for *E. ewingii*) and two for *Rickettsia* (one for *R. rickettsii* and one *R. felis*). None of these samples were previously tested for *Ehrlichia* or *Rickettsia* species by PCR. Overall, the detection rate using TBDCapSeq was substantially improved over PCR. TBDCapSeq identified a pathogen in 43 samples vs. 23 using PCR, with four co-infections detected vs. zero by PCR.

**Table 2:** Pooling strategy and read output for TBDCapSeq and unbiased next generation sequencing (UNGS).

Pool	# Samples	# of raw reads	Average reads/sample	Min reads	Max reads	# of reads after primer trimming
Pool 1	34	4.70E+08	1.38E+07	8.20E+06	2.83E+07	4.10E+08
Pool 2	21	3.69E+08	1.76E+07	8.38E+05	1.36E+08	3.45E+08
Pool 3	35	4.62E+08	1.32E+07	8.78E+06	2.31E+07	4.06E+08
Pool 4	35	4.23E+08	1.21E+07	6.07E+06	2.89E+07	3.72E+08
Pool 5	34	5.33E+08	1.57E+07	9.61E+06	4.31E+07	4.83E+08
Reproducibility test	34	4.60E+08	1.35E+07	6.23E+06	2.10E+07	4.13E+08
UNGS*^a^*	35	4.84E+08	1.38E+07	7.63E+06	2.53E+07	4.14E+08

a UNGS, unbiased next generation sequencing.

We did not identify any agents in the control group. When we retested a subset of our specimens (a combination of cases and healthy controls) with new library preparations, the data were 100% congruent to the results of the initial testing (Table 3).

**Table 3:** Reproducibility and comparative assay performance of TBDCapSeq.

	Normalized reads (per million filtered reads)											
	*Babesia microti*				*Borrelia burgdorferi*				*Rickettsia rickettsii*			
Sample*^a^*	Test 1	Test 2	UNGS*^b^*	qPCR	Test 1	Test 2	UNGS	qPCR	Test 1	Test 2	UNGS	qPCR
LYM-1308	1	0	0	Neg	0	0	0	Neg	**19**	**28**	0	ND*^c^*
LYM-1315	**10**	**14**	0	Neg	**3**	**2**	0	Neg	0	0	0	ND
LYM-1316	**126**	**172**	0	Neg	0	0	0	Neg	0	0	0	ND
LYM-1318	**107**	**128**	0	Neg	0	0	0	Neg	0	0	0	ND
LYM-1319	**28**	**14**	0	Neg	0	0	0	Neg	0	0	0	ND
LYM-1324	**85**	**82**	0	Neg	0	0	0	Neg	0	0	0	ND
LYM-1325	**1,093**	**1,020**	0	Neg	0	0	0	Neg	0	0	0	ND
LYM-1326	0	2	0	Neg	0	0	0	Neg	0	0	0	ND
LYM-1327	1	2	0	Neg	0	0	0	Neg	0	0	0	ND
LYM-1344*^d^*	0	0	0	Neg	0	0	0	Neg	0	0	0	ND

a Each sample was analyzed on two different TBDCapSeq assays (test 1 and test 2) with different library preparations, as well as by UNGS and qPCR; Samples in bold were identified as positive.

b UNGS, unbiased next generation sequencing.

c ND, not done.

d Healthy control.

Last, we examined a pool of 35 samples by UNGS to directly compare the sensitivity of TBDCapSeq to UNGS. This pool included samples identified by TBDCapSeq as being positive for *B. microti* (*N* = 6, including one PCR-positive sample), two with *B. burgdorferi* (*N* = 2), and one *R. rickettsii* (*N* = 1). Only the *B. microti* PCR-positive sample was identified by UNGS.

## Discussion

Diagnosis of early Lyme disease is a substantial clinical challenge (Aucott et al., 2009). The nonspecific signs and symptoms associated with Lyme disease, including the inconsistency of EM, combined with an often negative two-tiered serology and transient blood spirochetemia, all contribute to the difficulty in obtaining an accurate clinical and laboratory diagnosis. Co-infections can present additional complications. The primary vector of *B. burgdorferi*, *I. scapularis*, has been implicated in transmission of five different pathogens in the Northeastern United States (Tokarz et al., 2010, 2017; Sanchez-Vicente et al., 2019). Although *B. burgdorferi* remains the most prevalent tick-borne agent, we and others have demonstrated the increasing prevalence of *A. phagocytophilum* and *B. microti* in ticks from the Northeast (Clark et al., 2002; Tokarz et al., 2010; Aliota et al., 2014; Keesing et al., 2014; Goethert et al., 2018). Ticks infected with multiple pathogens are not uncommon, increasing the risk of human co-infections and the implication of co-infections for clinical management (Tokarz et al., 2010; Sanchez-Vicente et al., 2019).

Direct molecular tests can detect infection earlier than serologic tests, but their clinical utility for *B. burgdorferi* is limited by low sensitivity in blood, the most frequently tested specimen for TBD samples (Moore et al., 2016). The development of alternative molecular approaches, such as multi-locus PCR and electrospray ionization mass spectrometry (PCR/ESI-MS), and digital PCR, have led to improvements in sensitivity (Eshoo et al., 2012; Das et al., 2020; Mosel et al., 2020). In previous work, we demonstrated the improvement in sensitivity and genomic analyses of tick-borne agents that can be achieved using TBDCapSeq. In this study, we directly compared qPCR to TBDCapSeq as tools for molecular detection of tick-borne agents in human blood. We demonstrate that TBDCapSeq provides several advantages over qPCR. The presence of probes for all major agents enables simultaneous detection of all potential tick-borne infections in one assay. We identified co-infections through use of TBDCapSeq in four samples where qPCR failed to do so. Because TBDCapSeq provides genomic data, there is neither need for additional confirmatory tests nor assays for species identification. Finally, because the added cost of using capture probes can be <$20 per sample, it does not substantially add to the cost of NGS.

Our data also suggest that the sensitivity of TBDCapSeq is superior to qPCR. While all qPCR-positive specimens were detected by TBDCapSeq, we identified the presence of a tick-borne agent in 22 additional case samples that were PCR-negative. The deficiencies of molecular detection of *B. burgdorferi* can be partially addressed by TBDCapSeq. Of the 98 Lyme disease specimens tested from the early cohort, seven were positive by TBDCapSeq, while only one was detected by qPCR. While this is a substantial improvement, it is admittedly still not sufficient, particularly in light of the fact that 30% of these early cohort samples were serologically positive for a *B. burgdorferi* infection by the two-tiered assay. There are several ways sensitivity of TBDCapSeq could be improved further. Our tests were performed with extracts from only 400 μl of blood. Achieving higher sensitivity often requires testing of larger blood volumes (1–20 ml) because of the low spirochete burden present in the blood of infected patients (Das et al., 2020; Mosel et al., 2020). In addition, sensitivity may also be influenced on the blood fraction tested (Das et al., 2020). Finally, our pooling strategy for sequencing resulted in reduced read allocation for each sample. Reducing the number of specimens per assay would substantially increase sequencing depth and undoubtedly enhance sensitivity.

We found *B. microti* to be the most frequent co-infecting agent, present in 16 out of 98 early cohort samples (Table 1). In the Northeast, and especially on Long Island, NY, United States (where the majority of the cohort originated), *B. microti* is typically the second most common pathogen present in ticks. In nymphs, infection rates with *B. burgdorferi* on Long Island range from 16 to 37%, while infection rates with *B. microti* range from 9 to 25% (Tokarz et al., 2017; Sanchez-Vicente et al., 2019; NY-DOH, 2021). In one study, we found that 25% of *B. burgdorferi* infected nymphs were also *B. microti* positive (Tokarz et al., 2017). Serological findings also indicate that coinfection with *B. burgdorferi* and *B. microti* is not uncommon in humans (Krause et al., 1996; Martinez-Balzano et al., 2015; Zaiem et al., 2016; Wormser et al., 2019). In areas endemic for both diseases, 66% of Lyme disease patients also presented IgM and IgG antibodies to *B. microti* (Benach et al., 1985). On Long Island, 29% of individuals who tested positive for Lyme disease by the two-tiered test were also positive for *B. microti* (Curcio et al., 2016). Thus, babesiosis appears to be the most common concurrent TBD with Lyme disease.

A limitation of our analyses is the lack of corroborating evidence of our positive TBDCapSeq results in the PCR-negative samples. In the early cohort, all our positive calls were made within the “case” samples, which is a strong indication of the validity of our findings. Nonetheless, we lack data from another assay that would support our results, particularly for the high number of *B. microti*-positive samples that were negative by qPCR. Serologic data for *B. microti* was not available but would also likely not be helpful as these were all early acute samples with trace evidence of *B. microti* parasitemia. In future work, a combined molecular and serologic examination of paired baseline and follow-up specimens that demonstrated seroconversion would more clearly illustrate the utility of TBDCapSeq for early detection of tick-borne agents. The lack of corroboration of our findings had a direct effect on the establishment of our thresholds for the agents detected. For *B. microti*, the positivity threshold was higher than for bacterial agents on our assay. Even before normalization, the total read counts for all bacterial pathogens in control and clinical specimens (except positives) were typically zero. This was occasionally not the case for *B. microti*. In the clinical specimens, the clear *B. microti* positive samples contained hundreds to millions of normalized *B. microti* reads per sample. When we evaluated the 25 negative control samples, all contained less than one *B. microti* read per million of primer-trimmed reads, with the majority (18 out of 25) containing zero total reads according to our desired length and homology criteria. However, we sporadically recorded an elevated *B. microti* read count in some of the clinical specimens, albeit at a very low level (between 10 and 50 total reads per sample). While it is possible that these specimens may in fact have been very low positives, we cannot exclude other possibilities that involve technical or bioinformatics errors. Thus, we established a criterion well above the threshold required for our controls to add confidence in our positivity calls.

Within the past decade, NGS has moved from a fringe and complicated niche technique to one that has revolutionized biomedical research. NGS is used for a wide range of applications, including genomics, epigenetics, transcriptomics, oncology, personalized genome medicine, and pathogen detection and discovery. This has coincided with technological advancements that have led to a decrease in costs, labor, and length of time required for data generation and analyses. NGS has also begun to gain prominence in clinical microbiology and public health. The advent of portable sequencers has created a potential to establish NGS as a field deployable frontline platform. With minimal upfront processing and requirement of only a laptop, handheld sequencers are significantly faster and less expensive than conventional benchtop instruments. The addition of enrichment techniques such as TBDCapSeq to these versatile sequencers can enable pathogen detection and discovery at speeds unprecedented for other NGS platforms. Undoubtedly, further innovations will spur the use of this technique as a diagnostic tool and perhaps even as point-of-care tests in the future.

## Data Availability Statement

The datasets presented in this study can be found in the NCBI Sequence Read Archive. The names of the repository and accession numbers can be found at BioProject ID PRJNA796231 and accessions SAMN24835356-SAMN24835513.

## Author Contributions

RT and EH: conceptualization. RT: funding acquisition. TT, AG, VK, CG, and RT: methodology. SS-V, TT, AG, VK, and RT: investigation. SS-V, KJ, VK, and RT: formal analysis. EH, WIL, and RT: resources. SS-V and RT: validation and writing. All authors have read and approved the final manuscript.

## Funding

This project was funded by a grant to RT from the Steven & Alexandra Cohen Foundation.

## Conflict of Interest

The authors declare that the research was conducted in the absence of any commercial or financial relationships that could be construed as a potential conflict of interest.

## Publisher’s Note

All claims expressed in this article are solely those of the authors and do not necessarily represent those of their affiliated organizations, or those of the publisher, the editors and the reviewers. Any product that may be evaluated in this article, or claim that may be made by its manufacturer, is not guaranteed or endorsed by the publisher.
